# Supplementary material for: Launching of hyperbolic phonon-polaritons in h-BN slabs by resonant metal plasmonic antennas
Source: Nat Commun. 2019 Jul 19;10:3242. doi: 10.1038/s41467-019-11143-7 (PMC6642108; doi:10.1038/s41467-019-11143-7)
Supplement: Supplementary file 1 — Supplementary Information [file 41467_2019_11143_MOESM1_ESM.pdf]

Supplementary Information

Launching of hyperbolic phonon-polaritons in h-BN slabs by resonant metal plasmonic antennas

P. Pons-Valencia et al.

Supplementary Note 1: Procedure for smoothing the experimental spectra and comparing them to the simulation.

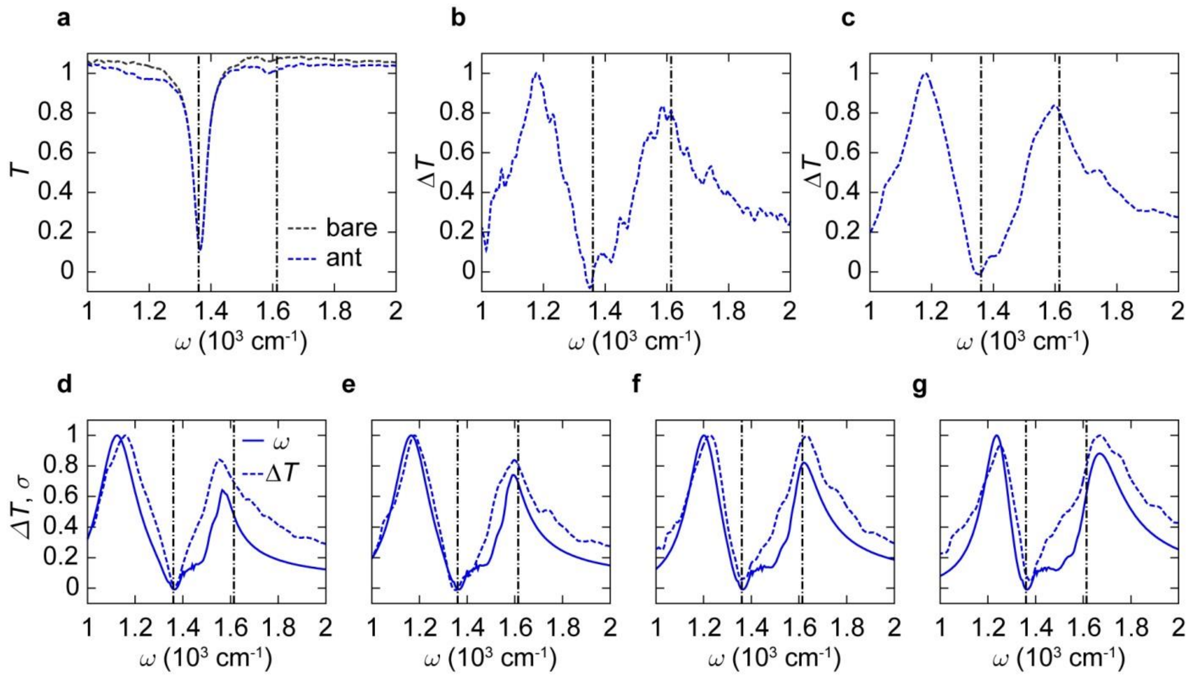

***Supplementary Figure 1: Smoothing of the FTIR spectra and its comparison with full-wave simulations**. (a) Transmission spectrum, T, for 2290 nm length antenna on h-BN slab (dashed blue line) and the one for the bare h-BN slab, T_0 (dashed black line). The vertical dashed lines indicate the frequencies of TO and LO phonons (low and high frequencies, respectively). (b) Difference transmission spectrum, $\Delta T = T_0 - T/\max(T_0 - T)$. (c) Difference transmission spectrum smoothed by the moving average method using 25 elements. (d-g) Comparison between the experimental ΔT and simulated extinction cross section, σ, for antennas of the following lengths: (d) 2400 nm, (e) 2290 nm (f) 2070 nm and (g) 1960 nm.*

We have performed the Fourier-transform infrared spectroscopy (FTIR) transmission experiments for the bare h-BN flake (with a thickness of 55 nm) and for the same flake with the gold rod antennas on top (with a rod thickness of 50 nm and a width of 175 nm). The experimental spectrum obtained from the bare h-BN flake (dashed black line in Supplementary Figure 1a) and the one obtained from bare h-BN flake with a gold rod antenna (of 2290 nm length) on top (dashed blue line in Supplementary Figure 1a) are very similar, and thus the effect of the antenna is difficult to distinguish. To better represent the effect of the antenna, we subtract and normalize the spectra, $\Delta T = (T_0 - T)/\max(T_0 - T)$ (Supplementary Figure 1b), where T_0 denotes the transmission through the h-BN flake, and T denotes the transmission through the h-BN flake with the antenna on top. The difference

transmission spectra show the two peaks, which are analyzed in the main text and in Section S4 below. To remove the noise from the spectra, we perform a moving average over 25 elements (50 cm^{-1}), obtaining the smoothed spectra shown in Supplementary Figure 1c. The averaged difference transmission spectra are then compared with the simulated extinction cross section, σ, for antennas of different lengths (see Methods). The experiment (Supplementary Figure 1d-g, solid lines) and theory (Supplementary Figure 1d-g, dashed lines) are in an excellent agreement. The positions of the peaks (red and blue squares in Fig. 2a) are found from the smoothed experimental spectra.

Supplementary Note 2: Dispersion and reflection phase of the waveguide mode propagating along the infinite gold rod on the h-BN slab

We interpret the measured resonances in the antenna as constructive interference of multiple reflections of the waveguide modes from the edges of the antenna (Fabry-Perot resonances). To that end we found both the wavevector, k_w, and reflection phase, φ, of the mode propagation along the infinite gold rod on the h-BN slab by means of full-wave simulations. The calculated wavevector as a function of frequency, $k_w(\omega)$, is shown in Supplementary Figure 2a. At low and high frequencies the dispersion $k_w(\omega)$ tends to the wavevectors of the mode of a gold waveguide over an isotropic slab with the dielectric permittivities $\epsilon_\perp(0)$ and $\epsilon_\perp(\infty)$ respectively. Inside the Reststrahlen band the dispersion of the mode shows a back-bending behavior, which is associated with high absorption of the mode. In the frequency range of the back-bending, the difference transmission spectra for antennas of different lengths show an anti-crossing feature (see Fig 2a). Notice that in some frequency ranges, due to the strong losses (and, in particular, due to the radiation losses) we have not been able to find the solutions for $k_w(\omega)$ (so that the curve Fig 2a has discontinuities).

In order to compare the mode dispersion with the results of the measurements, we find the reflection phase of the mode propagating in a truncated waveguide. For that we define the vertical electric field of the mode travelling along the semi-infinite waveguide (the region $x < 0$) as:

$$\boldsymbol{E_z}(x, y, z) = \left(\boldsymbol{e^{ik_w x}} + \boldsymbol{r_E e^{-ik_w x}}\right)\boldsymbol{F}(y, z), \qquad (1)$$

where $x = 0$ is the end of the semi-infinite gold rod and $F(y, z)$ accounts for the transverse field distribution of the waveguide mode, and the term $r_E e^{-ik_w x}$ in the parenthesis accounts for the reflection from the end of the waveguide ($x = 0$). The reflection coefficient can be written as $r_E = |r_E| e^{ik_w \varphi}$, where φ is the reflection phase. We calculate the phase by means of full-wave simulations. By subtracting the incident field from the total field given by Eq. (1), the reflection coefficient r_E is obtained. The extracted phase, $\varphi(\omega)$, is shown in Supplementary Figure 2b (we show $\varphi(\omega)$ only for those frequencies for which we managed to reliably separate the mode reflected at the end of the waveguide from the total

field). The reflection phase $\varphi(\omega)$ presents values around $\frac{3\pi}{12}$. By plugging in the average value of the phase, $\phi = \frac{3\pi}{12}$, into the Fabry-Perot resonance condition, $L \cdot k_w(\omega) + \varphi(\omega) = \pi$ (with L being the antenna length), we obtain the relation between the frequency and inverse antenna length (solid blue line in Fig. 2a), presenting an excellent agreement with the maxima in the difference transmission spectra (bright features in the colorplot in Fig. 2a).

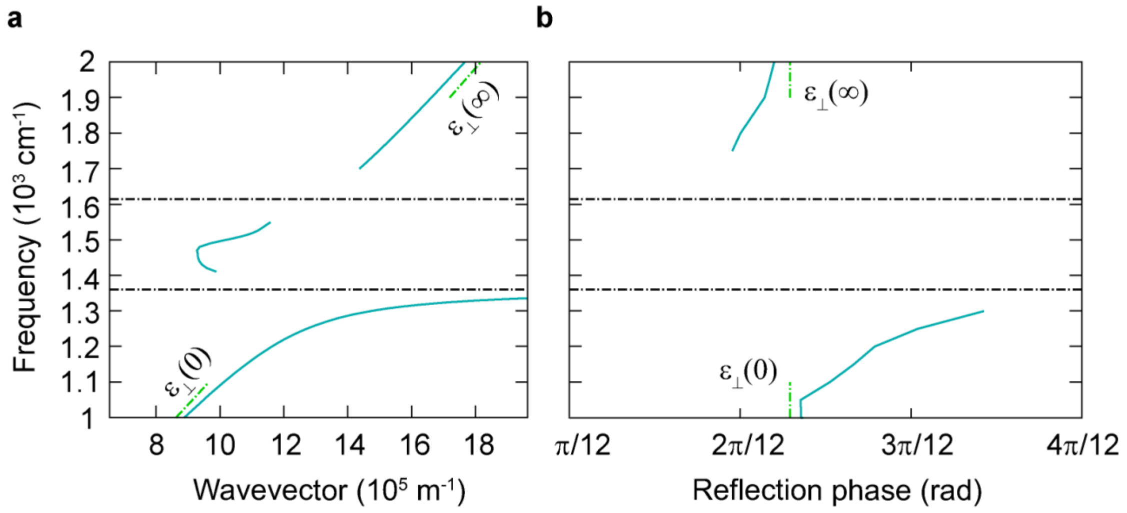

Supplementary Figure 2: Calculated wavevector and reflection phase of the waveguide mode of the gold rod on the h-BN slab*. (a) Wavevector of the waveguide mode, k_w, as a function of frequency. The waveguide presents an infinite gold rod (width 175 nm, height 50 nm) on top of the 55 nm-thick h-BN slab. (b) Reflection phase of the mode, $\varphi(\omega)$, found for the semi-infinite waveguide. The horizontal lines in (a) and (b) indicate the TO phonon (low frequency) and the LO phonon (high frequency), respectively. The dashed green lines trace the dispersion of the mode of the gold rod on the h-BN slab with the perpendicular permittivity, $\epsilon_\perp$, set as $\epsilon_\perp(\infty)$ and $\epsilon_\perp(0)$ at high and low frequencies, respectively.*

Supplementary Note 3: Polariton launching efficiency by the resonant antennas

Our nanoimaging experiments and numeric simulations demonstrate that resonant gold antennas can be efficient launchers of hyperbolic phonon-polaritons in h-BN slabs (See Fig. 5d and Fig. 6). We extend the analysis presented in Fig. 6, comparing the extinction cross section, σ, with the launching cross section, σ_{M0}, the launching cross section normalized to the geometric area of the antenna, $\sigma_{\mathrm{M0}}/S_{\mathrm{a}}$, and the field enhancement on the bottom face of the antenna. Namely, in Supplementary Figure 3 we present the calculated σ (panel a), σ_{M0} (panel b), $\sigma_{\mathrm{M0}}/S_{\mathrm{a}}$ (panel c), and the electric field enhancement (panel d) of the nanorod gold antenna on the h-BN slab as a function of frequency and inverse antenna length, $1/L$ (colorplots).

For a large part of frequencies within the Reststrahlen band, both normalized and non-normalized σ_{M0} clearly present an optimum in $1/L$. Both optima positions are blueshifted compared to the Fabry-Perot resonant condition. The optimum in σ_{M0} correlates well with the electric field enhancement on the bottom face of the antenna (Supplementary Fig. 3d)

In Supplementary Figure 4 we compare the normalized launching cross section, $\sigma_{\mathrm{M0}}/S_{\mathrm{a}}$, of the antennas with the non-resonant launchers: an infinitely long gold stripe (dashed blue curve) and a gold disk (dashed black curve) with the geometric area, S_{d}, equal to the geometric cross-section of the antenna, S_{a}. The resonant antenna shows about one order of magnitude better launching efficiency (reaching $\sigma_{\mathrm{M0}}/S_{\mathrm{a}}\sim 4$) compared to both metal disks ($\sigma_{\mathrm{M0}}/S_{d}\sim 0.7$ in the maximum, which is consistent with Ref. 1) and metal stripes ($\sigma_{\mathrm{M0}}/S_{\mathrm{stripe}}\sim 0.25$ in the maximum).

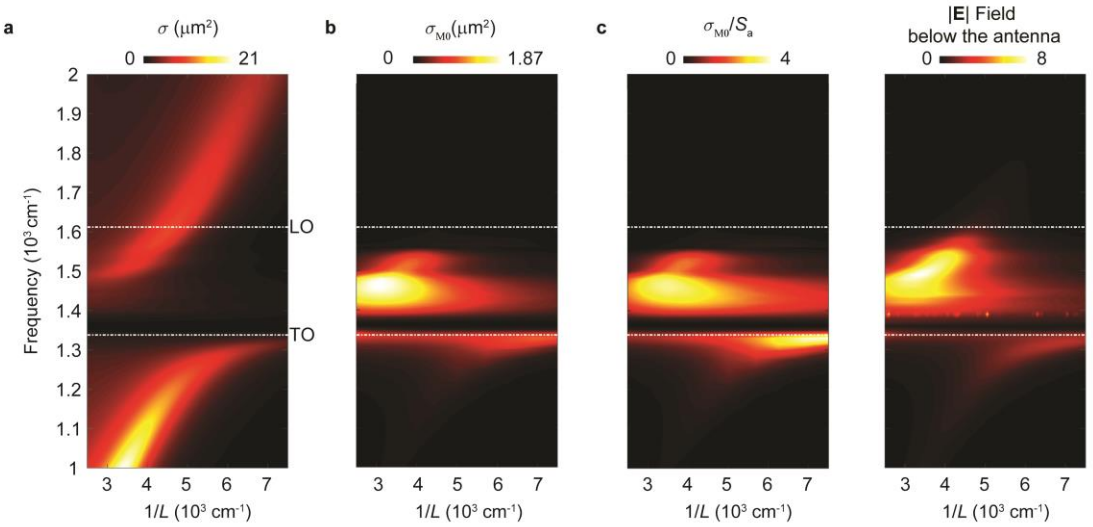

Supplementary Figure 3: Simulated extinction and launching cross sections. *(a) The extinction cross section as a function of the inverse antenna length,* $1/L$*, and frequency,* ω*. (b) The launching cross section as a function of* $1/L$ *and* ω*. (c) The launching cross section normalized to the antenna's geometric cross section, as a function of* $1/L$ *and* ω*. (d) The electric field enhancement, calculated on the bottom face of the antenna, as a function of* $1/L$ *and* ω*. The thickness of the h-BN layer is 55 nm and all the antennas have the thickness of 50 nm and the width of 175 nm. The horizontal white lines in (a)-(d) mark the positions of the TO and LO phonons.*

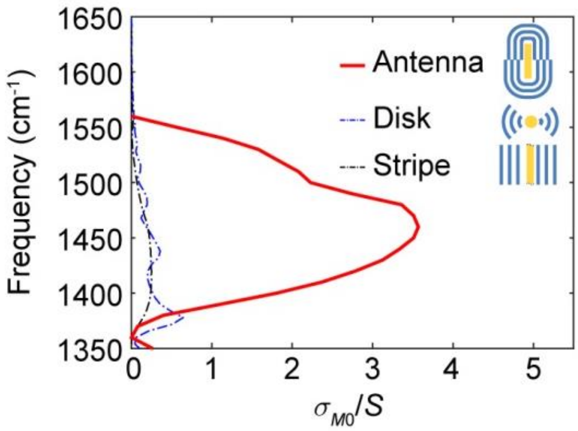

Supplementary Figure 4: Simulated launching efficiency of the resonant antenna compared with that of other launchers. *The normalized launching efficiency for the antenna of the length L=2.29μm (red line), for the disk with the same geometric cross-section as that of the antenna (dash-dot blue line) and for the stripe with the same width and height as those of the antenna (black dash dotted line). The thickness of the h-BN layer is 55 nm, while all the metallic structures have a thickness of 50 nm. The width of the stripe and the antenna is 0.175 μm.*

Supplementary Note 4: Model for strongly coupled classical harmonic oscillators

In order to analyze the difference transmission spectra of the resonant Au antennas on top of h-BN slabs, ΔT, we phenomenologically describe the coupling of the antenna modes and the phonon polaritons via the model of coupled classical harmonic oscillators. To that end, we start with the equations of motion (for the displacement, x) of each oscillator:

$$\begin{matrix} \ddot{x}_{HPhP} + \gamma_{HPhP}\dot{x}_{HPhP} + \omega_{HPhP}^2 x_{HPhP} - 2g\omega x_{SPP} = F_{HPhP} \\ \ddot{x}_{SPP} + \gamma_{SPP}\dot{x}_{SPP} + \omega_{SPP}^2 x_{SPP} - 2g\omega x_{HPhP} = F_{SPP} \end{matrix} \quad ,(2)$$

where ω_{HPhP} , ω_{SPP} represent the resonance frequencies, γ_{HPhP} , γ_{SPP} the damping ratios, corresponding to HPhP and SPP modes respectively, in the realistic electromagnetic problem. The coupling strength is g. In the right-hand side of Eq. (2), F_{HPhP} and F_{SPP} are the driving forces of the oscillators. Assuming a temporal dependence $e^{-i\omega t}$, Eq. (2) becomes

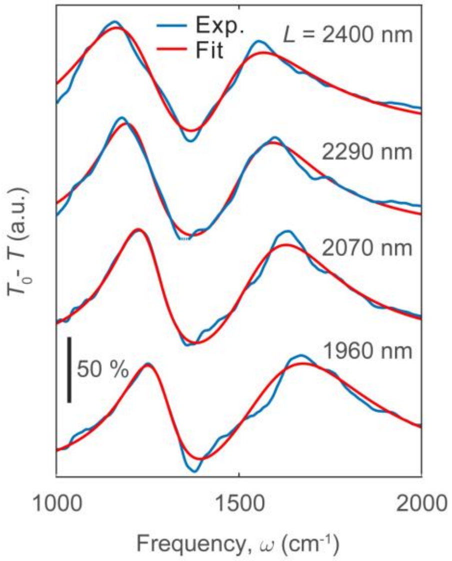

Supplementary Figure 5: Fitting the experimental extinction spectra with the model of two oscillators. *Experimental extinction spectra of antennas of the length L = 2400, 2290, 2070 and 1960 nm from top to bottom (solid blue lines). The solid red curves represent the fitting to the classical oscillators model, given by Eq. (6).*

$$-\omega^2 x_{HPhP} - i\gamma_{HPhP}\omega x_{HPhP} + \omega_{HPhP}^2 x_{HPhP} - 2g\omega x_{SPP} = F_{HPhP}$$
$$-\omega^2 x_{SPP} - i\gamma_{SPP}\omega x_{SPP} + \omega_{SPP}^2 x_{SPP} - 2g\omega x_{HPhP} = F_{SPP}. \quad (3)$$

We solve the system for the positions x_{HPhP} and x_{SPP}, obtaining

$$\begin{pmatrix} x_{HPhP} \\ x_{SPP} \end{pmatrix} = A^{-1} \begin{pmatrix} F_{HPhP} \\ F_{SPP} \end{pmatrix} \quad (4)$$

where $A = \begin{bmatrix} -\omega^2 - i\gamma_{HPhP}\omega + \omega_{HP}^2 & -2g\overline{\omega} \\ -2g\overline{\omega} & -\omega^2 - i\gamma_{SPP}\omega + \omega_{SPP}^2 \end{bmatrix}$ with $\overline{\omega} = \frac{\omega_{HPhP} + \omega_{SPP}}{2}$.

The normalized difference spectra then can be approximated as[2]

$$\Delta T \propto \langle F_{SPP} \cdot \dot{x}_{SPP} + F_{HPhP} \cdot \dot{x}_{HPhP} \rangle \quad (5)$$

which then using Eq. (4) can be explicitly written as

$$\Delta T \propto \omega F_{HPhP}^2 \mathrm{Im}\left[\frac{-\omega^2 - i\gamma_{SPP}\omega + \omega_{SPP}^2}{\det A}\right] + 2\omega F_{HPhP} F_{SPP} \mathrm{Im}\left[\frac{2g\omega}{\det A}\right]$$
$$+ \omega F_{SPP}^2 \mathrm{Im}\left[\frac{-\omega^2 - i\gamma_{HPhP}\omega + \omega_{HPhP}^2}{\det A}\right]. \quad (6)$$

We use Eq. (6) to fit the difference transmission spectra obtained experimentally, as illustrated in Supplementary Figure 5. The frequencies of the eigenmodes of the system, $\omega^{\pm}$, obtained from the condition $\det A = 0$, read

$$\omega^{\pm} = \frac{1}{2}(\omega_{HPhP} + \omega_{SPP}) \pm \frac{1}{2}\mathrm{Re}\left[\sqrt{4|g|^2 + \left[\delta + i\left(\frac{\gamma_{SPP}}{2} - \frac{\gamma_{HPhP}}{2}\right)\right]^2}\right]. \quad (7)$$

In Supplementary Figure 6a we show the positions of the peaks, ω^{+} and ω^{-}, extracted from the experimental data (circular and square symbols), the maxima of the spectra corresponding to Eq. (6) (thin dashed lines), and the $\omega^{\pm}$ calculated according to Eq. (7) (solid lines). In the same panel, the values of ω_{HPhP} and ω_{SPP} (frequencies of the uncoupled oscillators) are shown by the thick dashed lines.

As previously reported[2], the splitting between the eigenfrequencies, $\omega^{\pm}$, found form Eq. 7 is smaller than that found from the positions of the maxima of the spectra given by Eq. 6.

The extracted values of γ_{HPhP} , γ_{SPP} and g are shown in Supplementary Figure 6b. Recall that two typical conditions for strong coupling can be defined as[3,4] $C_1 = \frac{|g|}{(\gamma_{HPhP} - \gamma_{SPP})} > 0.25$ and, the more restrictive one, $C_2 = \frac{|g|}{(\gamma_{SPP} + \gamma_{SPP})} > 0.25$. According to the results presented in Supplementary Figure 6b, both conditions are fulfilled, as illustrated in Supplementary Figure 6c by plotting C_1 and C_2, as a function of frequency.

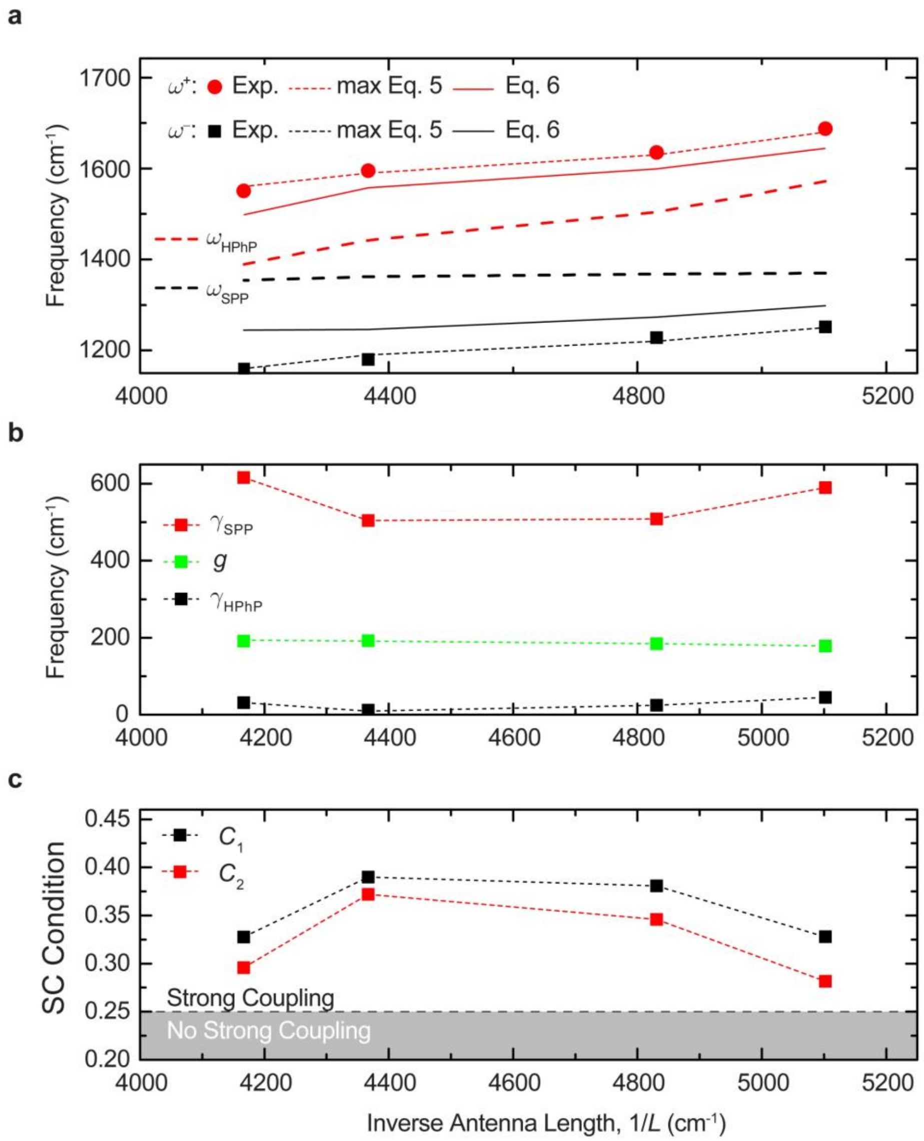

Supplementary Figure 6: Analysis of the coupling between SPPs and HPhPs according to the classical oscillators model. *(a) Frequencies of the peaks,* ω^{+} *and* ω^{-} *according to the experiment (red and black symbols), according to the fitting by Eq. (6) (thin dashed lines), and its eigenfrequencies according to Eq. (7) (thin solid lines). Thick dashed lines correspond to the fitted values of* ω_{HPhP} *and* ω_{SPP}*. (b) The fitted values of the coupling strength,* g*, and the dissipative terms,* γ_{SPP} *and* γ_{c}*, obtained from the experimental data. The dashed lines are guides to the eye. (c) Conditions for the strong coupling,* C_1 *and* C_2 *(black and red symbols, respectively; lines are a guide to the eye). The white background indicates the zone where the strong coupling regime is reached.*

Supplementary References

1. Dai, S. et al. Efficiency of Launching Highly Confined Polaritons by Infrared Light Incident on a Hyperbolic Material. *Nano Letters* **17**, 5285-5290 (2017).

2. Autore, M. et al. Boron nitride nanoresonators for phonon-enhanced molecular vibrational spectroscopy at the strong coupling limit. *Light: Science &Amp; Applications* **7**, 17172 (2018).
3. Törmä, P. & Barnes, W.L. Strong coupling between surface plasmon polaritons and emitters: a review. *Reports on Progress in Physics* **78**, 013901 (2015).
4. Melnikau, D. et al. Rabi Splitting in Photoluminescence Spectra of Hybrid Systems of Gold Nanorods and J-Aggregates. *The Journal of Physical Chemistry Letters* **7**, 354-362 (2016).
